# Multi-Directional Universal Energy Harvesting Ball

**DOI:** 10.3390/mi12040457

**Published:** 2021-04-19

**Authors:** Ryan G. Hall, Reza Rashidi

**Affiliations:** Department of Mechanical and Electrical Engineering Technology, State University of New York, Alfred State College, Alfred, NY 14802, USA; hallrg@alfredstate.edu

**Keywords:** energy, harvester, electromagnetic, compression, multi-directional, universal, silicone, sphere, core, shell, ball

## Abstract

This paper discusses the development of a multi-directional, universal, electromagnetic energy harvester. The device is a ball consisting of two parts: a rigid spherical core with internal tubes, coils and magnets, and a flexible silicone-based shell holding a carrier fluid. The multi-directional aspect of the design comes from the device’s spherical shape. The harvester generates energy when subject to compressive force, by moving fluid through a tube, pushing a permanently magnetized ball through a coil wound around the tube. A combination of 3-D printed PLA plastic and molded silicone was used to produce a prototype. The energy harvester can be utilized in applications where there is an oscillating compression and it is not limited to certain applications due to its universal ball shape. It was tested at five different frequencies between 4–15 Hz on its four different outer sides producing electricity at a range of 17 to 44 mV.

## 1. Introduction

As technology advances, more electronic devices will require new and innovative power generating solutions. When looking at how manufacturing is advancing, it is safe to assume that technology is being fit into more compact, and odd places. For example, people carry multiple devices each day such as a phone, watch, key fob, or health monitoring systems. Another need is to develop long lasting power in spaces where the use of batteries is not feasible. A traditional solution to this problem would be to limit the amount of electrical power consumed [1], but if that design limitation is removed, the possibilities of data monitoring are endless. Energy harvesting devices can be utilized in parallel with the electronics in order to enable a theoretically, unlimited power supply [2]. For instance, a device that can harvest energy from motion could be used in scientific studies of endangered animals to be implemented in the trackers placed on the animal. Instead of tracking an animal for the life of the battery it can be tracked throughout its whole life providing valuable data to researchers [3,4]. This type of tracking device would be difficult and sometimes impossible to recharge. In this case, the possibility of continual data monitoring would require energy being harvested from motion. Motion from the human body [5], animals [6], or mechanical equipment [7] can be used to charge batteries [8,9] or power objects, such as wearable devices [10,11], tactile sensors [12,13], and biomedical devices [14,15]. This motion can be generated by rotational [16,17,18,19], linear [20,21], vibrational [22,23,24], or forced fluid induction [25,26,27,28] systems.

Energy harvesting from a moving object is done in various ways: using piezoelectric [29,30,31], electrostatic [32,33], electromagnetic [34,35,36], and triboelectric [37,38] methods. Piezoelectric elements, when vibrated, produce electricity. This vibration can be produced by an applied force or a magnetic attraction. This method has been developed to work with various natural and mechanical devices [39,40,41], as well as on the human body [42], [43]. In addition, piezoelectric materials can be deposited as a film over acrylic sheets used in fluid flow devices producing energy due to vibration from turbulence [44]. Another way of producing energy from motion is electrostatically where a variable capacitor is utilized to generate energy. In this device, one of the capacitor’s electrodes is stationary while the other one is moved due to an external force, thus changing capacitance and producing energy [45,46]. In addition, the triboelectric method is used to harvest energy through a charging process in friction. The energy conversion from motion to electricity is performed by friction between two polymeric materials with different triboelectric properties, charging an interface layer between two materials and generating energy [47,48]. Furthermore, energy harvesting can be done through electromagnetism where there is a motion between a magnet and a coil of wire [49,50]. As an example, an engine can drive the generator of an alternator, producing electricity required in a car. Various devices that use magnetic concepts have been demonstrated by this group [31,36,51,52,53,54,55,56].

An eccentric electromagnetic energy harvester consisting of pendulum rotor with alternative distributed magnets in the outer side was demonstrated. The results showed the device could work in very low frequencies with an output power suitable for low-power wireless sensor nodes [57]. A hybrid piezoelectric and electromagnetic energy harvester was demonstrated and the effects of several factors including load resistance, electromechanical coupling, mechanical damping, coil parameter, and size on the device was studied theoretically and experimentally. It was shown that the linearized electromagnetic coupling is the best fit for low excitation acceleration and hybrid mechanisms would widen the frequency bandwidth and output power [58]. 

A magnetically forced energy harvester consisting of two moving magnets supported by elastic beams was demonstrated. An energy localization and mechanical nonlinearity was produced as a result of large movement of the beams. The results showed improvements in the harvested power and frequency bandwidth compared with nonlinear devices [59]. An archetypal energy harvester was used to nonlinearly convert medium vibration into electrical power using electromagnetic induction. A mathematical model was presented to show the effect of circuit inductance on the resonance frequency of the device [60]. A nonlinear electromagnetic energy harvester was developed to convert vibration into electrical energy. The harvester consisted of moving magnets held by elastic springs to create repulsive magnetic force and low mechanical damping. This structure functionalized a localization phenomenon and resulted in an improvement in the device performance [61]. The design and model of an energy harvester utilizing magnetic levitation to generate oscillation was demonstrated. The theoretical and experimental results showed that the design produces large oscillations following Duffing’s equation for relative displacements under both static and dynamic loads [62].

Few papers have been shown to utilize a ball, either magnetic or nonmagnetic in their electromagnetic energy harvesting devices. In a developed energy harvester, a nonmagnetic ball freely moves within a cylinder and hits two magnets suspended on helical springs, causing a vibration with higher frequencies and inducing a voltage in the coil round the cylinder due to the relative motion between the magnets and coils [63]. An electromagnetic energy harvester utilizes a nonmagnetic ball which can move and impact the parabolic top surface of a magnet mass, causing a vibration and relative motion between the magnet and a coil, inducing a voltage across the coil ends [64]. A miniaturized energy harvesting device using two guided magnets and a freely movable non-magnetic ball was demonstrated. The metal ball between the two magnets in a cylinder will move and transfers the human motion to compression springs, causing a relative motion between the coil and magnet, and inducing a voltage in the coil [65]. Another electromagnetic energy harvester consisting of a magnetic ball moving on a two-dimensional surface of a goblet-like structure allowed electricity generation in the coils embedded on the outer surfaces of the structure [66]. A body-worn energy harvester converting motion energy into electrical power was demonstrated. A magnetic ball moves within a spherical housing due to human motion, inducing a varying magnetic flux in the coils and generating a voltage across their ends [67]. 

A drawback to the aforementioned devices is that they are specifically designed for particular applications and their mechanical shape and design is only suitable for certain environments. Therefore, if one needs to utilize the device in another application, a redesign and re-fabrication is required. In addition, most of these energy harvesters are usually designed to be used in one direction. These factors make the existing devices expensive and usable for only limited applications. In the above example, this would mean that a different design of power generation would have to be developed for each individual animal, person or mechanical application. 

This paper presents a novel design of an energy harvester that overcomes these drawbacks. This energy harvester incorporates a forced fluid to push a ball-shaped magnet through a coil solenoid to generate power. Although the coil is placed in a certain direction in the design, a force on any side of the sphere shaped device causes movement of the fluid pushing, the magnet through the coil. This feature makes the energy harvester multidirectional. In addition, the device would be universal as the ball shape of the device has no preference on the application it is being used for. This paper discusses the design, fabrication, and characterization of the energy harvester. Effects of force frequency and changes in the location of the exerted force on voltage output are analyzed.

## 2. Materials and Methods

### 2.1. Design

**Figure 1:**
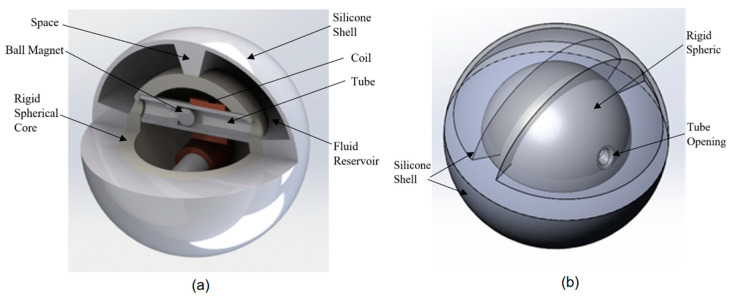
SolidWorks model of the energy harvester showing internal features (**a**) and the rigid spherical core and the silicone shell (**b**).

The energy harvester and its fabrication accessories were designed using SOLIDWORKS. The model of the device and its internal features are shown in Figure 1a. The device has a rigid spherical core 50 mm diameter made of Polylactic Acid (PLA) material consisting of two similar bonded halves. This diameter was selected as an optimum one. It should be large enough to ensure that there will be enough contact surface between the shell and external object (e.g., ground) and so enough fluid available to push the ball magnet through the coil while it should small enough to be movable. As shown in Figure 1a, each spherical half has a 5.1 mm diameter opening to allow for fluid flow into a tube. Each half encloses a tube with a 200-turn solenoid coil wound over the tube. The tube measures 10.2 mm in outer diameter and an inner diameter of 7.4 mm, giving a 6.4 mm diameter permanently magnetized ball enough tolerance to freely move through the tube. This magnet size was selected to ensure that it can generate enough induction in the coil while it is still light enough to avoid any resistance against fluid movement. The wall of the tube has a maximum thickness of 1.4 mm to ensure the coil is as close to the magnet as possible to produce the highest voltage for this size application. The tube also has a check spacer placed 26.0 mm in from its opening to allow fluid flow but to stop the ball from moving past the coil. This allows for the magnet to travel as much as 19.6 mm.

The rigid spherical core is enclosed within a silicone shell. A fluid reservoir is formed in the gap between the inner surface of the shell and the outer surface of the rigid spherical core. This distance was designed to be 12.0 mm in order to deliver enough displacement of the fluid in the reservoir to effect movement of the ball magnet when the outer surface of the shell is exerted upon by a flat surface. The outer and inner diameters of the shell are 76.2 and 73 mm, respectively, giving it a wall thickness of 1.6 mm which is optimal for the flexibility of fluid flow while maintaining durability from potential impact. As shown in Figure 1b, half of the shell is separated from the other half by a silicone spacer and each half is split equally with a center silicone rib of 5.1 mm chamfered up to 11.7 mm, allowing fluid flow only through the tube associated with that half. With this configuration, the shell reservoir is split to four regions. The overall size of the ball, as well as the dimensions of features, were selected based on the feasibility restrictions.

**Figure 2:**
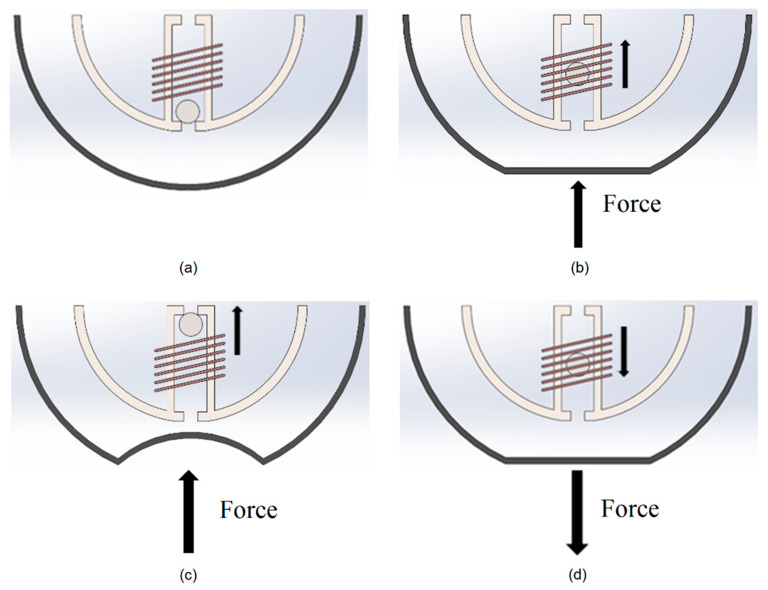
Working Principle of the energy harvester. The ball magnet initially falls in the bottom of the tube due to gravity (**a**). When the outer surface of the harvester shell is pressed, the reservoir is compressed displacing the fluid through the tube, pushing the ball magnet through the coil and inducing a voltage in the coil (**b**,**c**). When the force is taken away from the bottom side of the shell, the fluid and ball return to their original state downwards through the coil, inducing a negative voltage in the coil (**d**).

The working principle of the device is illustrated in Figure 2. The ball magnet initially falls in the bottom of the tube due to gravity (Figure 2a). When the outer surface of the harvester shell is pressed due to any external force, the reservoir is compressed displacing the fluid through the tube and pushing the ball magnet through the coil (Figure 2b,c). This movement of the ball magnet relative to the coil causes a change in the coil magnetic flux, inducing a voltage in the coil according to Faraday’s law of electromagnetism. When a magnet with its north to south axis aligned with the longitudinal axis of the coil moves though the coil, an emf voltage is generated in the coil due to magnetic induction. 

There is another reservoir on the other end of the tube in opposite side of the shell in which the flexible surface of the shell expands to accept the excess fluid that is displaced. When the force is taken away from the bottom side of the shell, the volume of the bottom reservoir expands again and the fluid returns to its original state due to the tensile force of the silicone in the upper shell and gravity (Figure 2d). The ball magnet then returns through the coil causing a second change in the magnetic flux, inducing a negative voltage in the coil. These two movements of the ball through the coil generate an alternative voltage cycle. 

The viscosity and type of fluid used in the energy harvester has a significant effect on the output. It was decided to use olive oil instead of water because the viscosity of the oil will account for the tolerancing inaccuracy between the diameter of the inner tube and the diameter of the ball magnet. A less viscous fluid, such as water, would allow the fluid to rush past the magnet and not to be able to push it through the coil.

### 2.2. Device Fabrication

**Figure 3:**
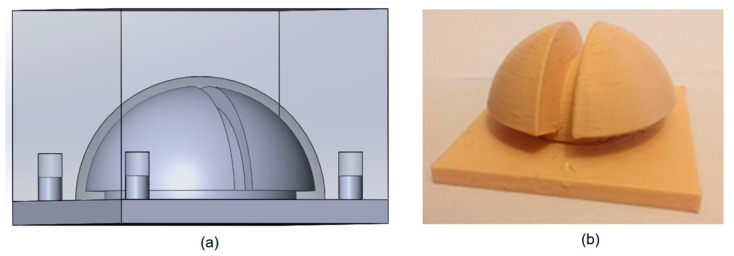
(**a**) SolidWorks model of the shell mold. (**b**) 3D printed mold used to fabricate two similar halves of the silicone shell.

The energy harvester was fabricated using 3D printing and molding. The rigid spherical core and its internal features were formed using a 3D printer with PLA material. This material made the harvester strong enough to resist possible impacts during the service. A mold was also fabricated using the 3D printer with PLA material and was used to make two halves of the silicone shell, as shown in Figure 3. A liquid soap was applied on the entire surface of the mold to make the surface hydrophobic and ensure easy removal of the shell after curing. The silicone epoxy was inserted into the mold and left overnight to cure. The halves of the silicone shell were carefully removed from the mold when cured.

**Figure 4:**
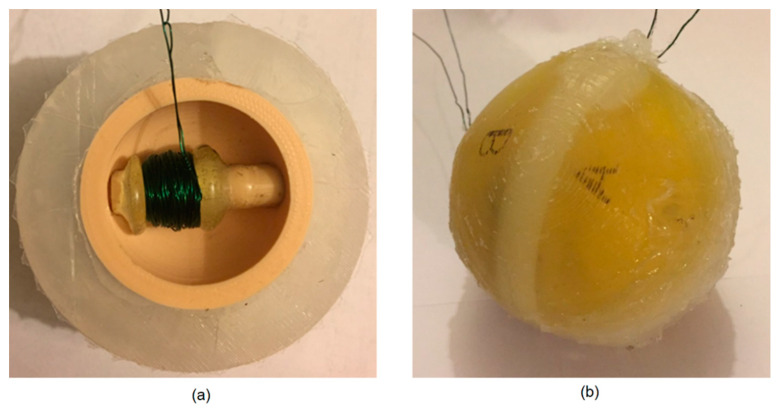
(**a**) Dissection of energy harvester body including the outer silicone shell, the rigid spherical core, coil, ball magnet, and tube. (**b**) Fabricated ball-shaped energy harvester. The two wires are for each coil in the core. The outer surface is divided to four sides and marked as A, B, C, and D for testing purposes.

The tube inside the rigid spherical core was wrapped with a 0.4 mm diameter wire to form a 200 turn, 12 mm long coil exactly in the middle portion, where the magnetic ball would travel. With the magnet inserted into the tube, it was then secured and sealed into the internal opening of the rigid spherical core using the silicone epoxy. One of the halves of the silicone shell was attached to the rigid spherical core using the silicone epoxy. The assembled half of the harvester body is illustrated in Figure 4a.

The entire process was repeated for the other half of the sphere producing two identical halves. In order to prevent fluid from leaking through the rigid core, its open center was filled with silicone epoxy. This made the fluid follow the one path from the depressed reservoir into the opposite reservoir through the tube. The two assembled halves were bonded together using the silicone epoxy to create the energy harvester, as shown in Figure 4b. 

### 2.3. Measurement

**Figure 5:**
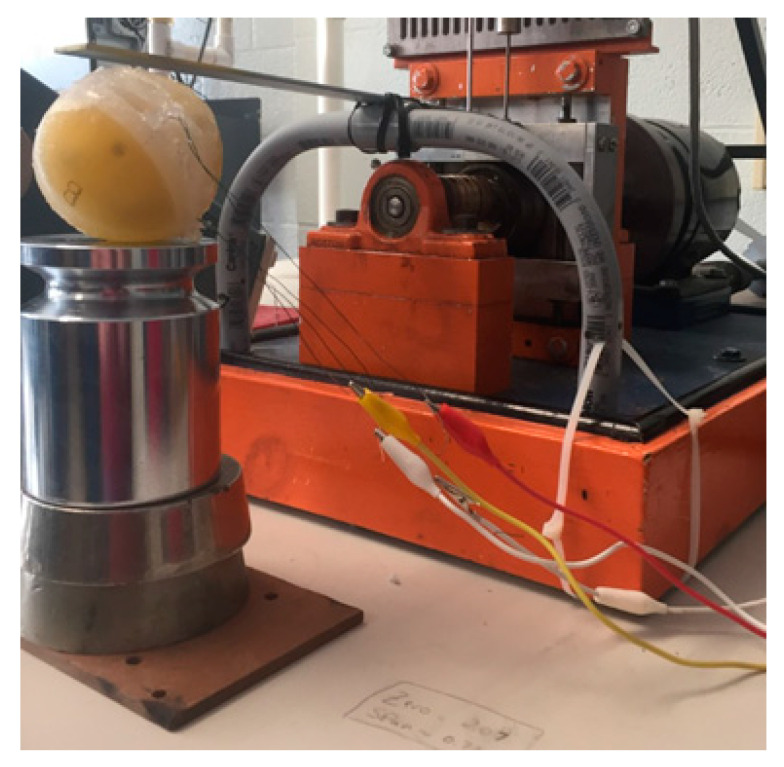
Variable speed Scotch Yoke apparatus with a custom setup used to test the energy harvester at different oscillating rates.

The device was tested using a variable speed Scotch Yoke apparatus which oscillates at different rates. To prepare the testing set up a steel cantilever beam was installed on the apparatus to achieve a proper displacement and fully depress the silicone shell on the harvester, as shown in Figure 5. The motor established a definite rotational velocity which was converted into a frequency.

The two wires of each coil protruding from the device were directly plugged into a data acquisition device (DAQ) to stream the readings. A National Instruments USB 6003 multifunctional DAQ was set up to measure the voltages as a function of time. A LabVIEW software was used to allow for the recording of generated voltages. In addition, the machine frequency was recorded using LabVIEW. 

The measurements were carried out every millisecond at six different oscillating rates between 4 and 15 Hz. This range of rates is available in many applications, such as appliances, bicycle, or car tire. The tests at rates lower than the range resulted in inconsistent and inaccurate readings. The reason could be that a slow depression of the shell caused the fluid to move slowly into the tube. As a result, the fluid was not able to push the ball magnet efficiently through the coil. When the tests were performed at faster rates, the voltage amplitudes significantly dropped. This could be due to the fact that the ball magnet did not get enough time to move up through the coil before the elasticity of the upper expanded silicone shell forced the fluid to move back to the bottom side of the tube. Another possibility might be that the high speed fluid could pass over the ball magnet and leak from the gap between the magnet and internal surface of the tube without being able to efficiently move the magnet up through the coil. These two factors could limit the travel span of the ball magnet through the coil, diminishing the voltage generation.

In order to determine how the energy harvester responds when different sides of the device are externally forced, the device’s outer surface was divided to four different sides, marked as A, B, C, and D. Each side has two lower and upper surfaces. All four sides were tested at various frequencies and the voltage of both coils corresponding to the two halves of the device were measured. Visually, the four sides of the ball from its external view are similar but, because of the orientation of the two internal tubes in the ball, each coil may respond differently due to random impact of the ball surfaces. The four sides of the ball are internally separated from each other. The sides are separated from each other by by a silicone spacer and a center silicone rib, allowing fluid flow only through the tube associated with that half. This will guarantee the highest fluid pressure and motion to the ball magnet when one of the surfaces is individually impacted.

As most applications require higher magnitudes of DC voltage, the voltages generated by the energy harvesting ball may not be directly usable. Thus, a power management circuit is required to be placed between the energy harvesting ball and the application load. The circuit may require a few basic components including an AC to DC rectifier and a booster device for increasing the magnitude of the harvested voltage to an acceptable level. Examples of a DC–DC booster device suitable for the harvester would be LTC3108-1 (Linear Technology) or the circuit presented in this paper [68]. An example for the AC to DC rectifier could be the electronics demonstrated in this paper [69]. A complete power management system suitable for the application is presented in this paper [70].

## 3. Results and Discussion

**Figure 6:**
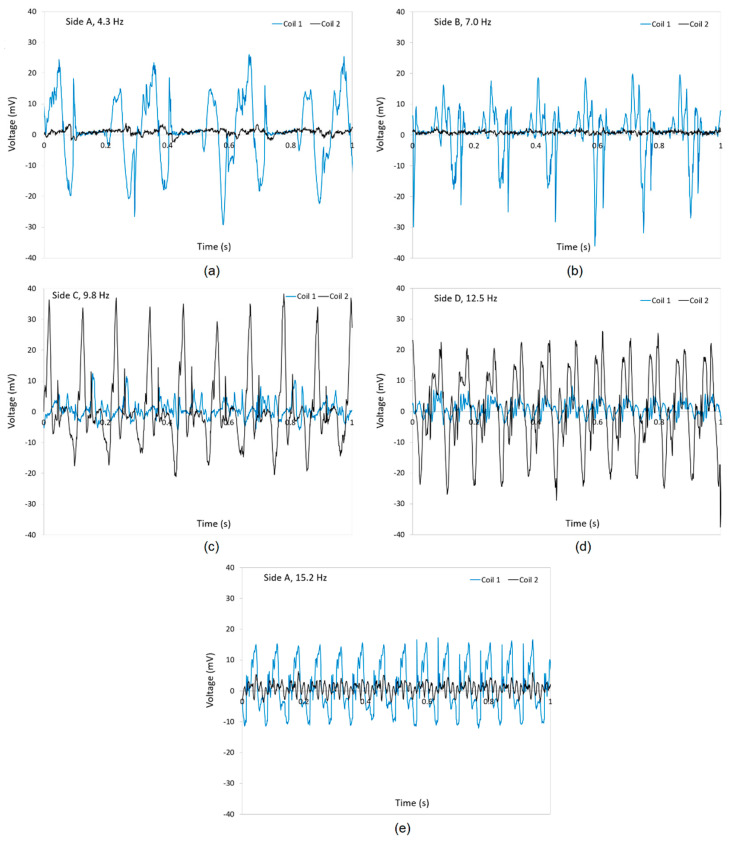
Generated voltages in coils 1 and 2 of the energy harvester as a function of time when (**a**) side A is subject to a compression at 4.3 Hz rate, (**b**) side B is subjected to a compression at 7.0 Hz rate, (**c**) side C is subjected to a compression at 9.8 Hz rate, (**d**) side D is subjected to a compression at 12.5 Hz rate, and (**e**) side A is subjected to a compression at 15.2 Hz rate.

Figure 6a–e show the generated voltage as a function of time at different frequencies of 4.3, 7, 9.8, 12.5, and 15.2 Hz for all four sides of the ball (A, B, C, and D). Each figure includes the voltage readings from both coils of the harvester. As shown in Figure 6a,b, the voltages generated in coil 1 are dominant compared with those induced in coil 2 when the harvester is compressed from sides A and B, while the voltages generated from coil 2 are significantly higher than the ones produced in coil 1 when the harvester is compressed from sides C and D, as shown in Figure 6c,d. This effect would be due to the orientation of the tubes and coils relative to each side. However, one can find that the device generates a high voltage either in coil 1 or coil 2 when compressed randomly from each of the four sides. This fact makes the harvester capable of harvesting energy in multiple directions.

When comparing the voltages in side A at 4.3 Hz (Figure 6a) with the ones in the same side at 15.2 Hz (Figure 6e), it is clear that the amplitude of the voltages in the main coil 1 slightly decreased. On the other hand, the voltages induced in coil 2 increased at 15.2 Hz. This effect in coil 2 could be due to the fact that the orientation of coil 2 affects the high speed of the fluid on the ball magnet while the speed of the fluid at low frequencies is not high enough to be able to move the ball magnet efficiently in an inclined coil 2.

Periodic times experimentally measured in a full cycle of the waveforms were used to calculate experimental response frequencies and compare with the excitation frequencies. The periodic time in full cycles of voltage was calculated from the times measured at each excitation frequency. These times were then converted into response frequencies. Table 1 presents the excitation and response frequencies and a deviation at each frequency. As expected, the response frequencies are close to excitation frequencies. The excitation frequency caused the fluid to push the ball magnet through the coil and the voltages induced in the coil are due to this motion. Therefore, it is expected to see similar frequencies. The deviation at each frequency can be due to resilience of the fluid and the delay in the movement of the ball through the coil.

On the other hand, the ball magnet which has two north and south poles may orient randomly in the coil when pushed by the fluid through the tube. As a result, the voltage amplitude depends on the orientation of the ball relative to the coil. It may induce voltages from zero to a highest possible in the coil. When the north-to-south axis of the ball magnet is along the longitudinal axis of the coil, a maximum voltage will be produced, however, when the north-to-south axis of the ball magnet is aligned perpendicular to the longitudinal axis of the coil, no voltage will be induced in the coil. Any other angle between these two axes will results in a voltage between zero to the highest possible voltage. As a result, the amplitude of the voltages generated in the coil will be randomly. 

**Figure 7:**
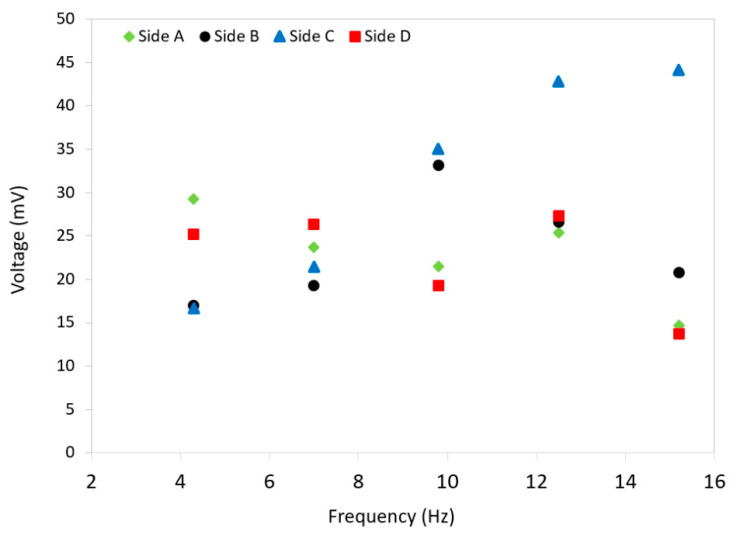
A comparison between the peak voltage amplitudes of all four sides of the harvester at different frequencies.

The average values of peak voltages were found across all frequencies for each side to determine what voltage could be achieved from each section of the device and how the energy harvester behaves when randomly compressed on any side. Figure 7 compares the peak voltage amplitudes as a function of frequency for all four sides of the harvester. As shown, the side C shows an increase in voltage amplitude from less than 20 mV as the frequency rises. There is a consistent voltage in all other sides at different frequencies. It is clear that all sides generate a consistent voltage 22 mV at 7.5 Hz. As a result, it can be discussed that the energy harvester would generate a consistent voltage from either coil 1 or coil 2, with a small variation when subject to a force from a random side. It is similar to the scenario that a ball can fall to ground from any random point when thrown up. This is an important aspect of the developed harvester as the user would not need to check the direction of the harvester when in use. It would supply almost a consistent energy.

It is to be noted that the maximum voltage in sides A and B comes from the coil 1 which is oriented to achieve the maximum voltage in the coil when those sides are compressed, while the maximum voltage is produced from coil 2 when either of sides C or D are compressed. There may be some differences in voltage readings from coil 1 and coil 2 due to errors in obtaining exact tolerances of the 3D printer when trying to make two exact tubes. In addition, the quality of winding the two coils might slightly affect the readings from the two coils. Furthermore, the two ball magnets might be slightly different due to errors in their manufacturing, even though it was assumed that the two magnets are similar. 

**Figure 8:**
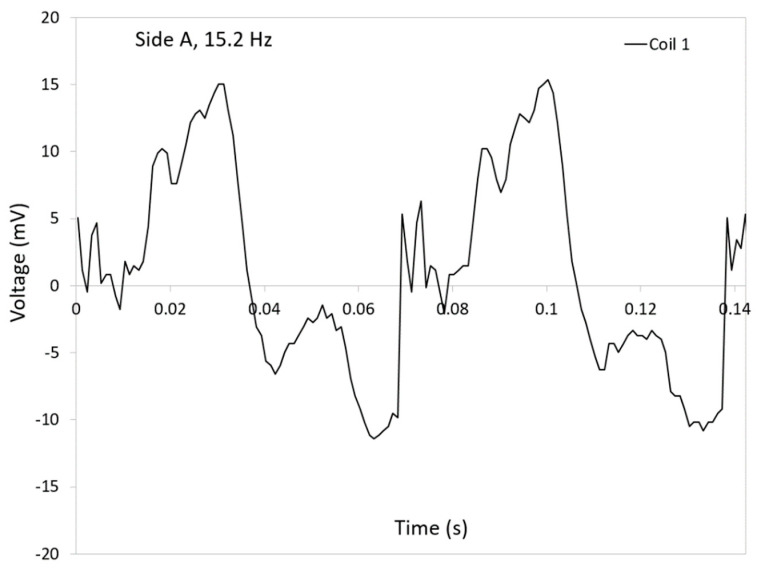
Expanded graphs of the generated voltages in coil 1 as a function of time when the side A of the harvester is subjected to a compression at 15.2 Hz rate.

Figure 8 illustrates the expanded graphs of the generated voltages in coil 1 when the side A is compressed at 15.2 Hz. The graphs show a clear sine wave corresponding the travel of the ball magnet back and forth inside the coil. The positive peak occurs when the ball magnet moves upwards while the negative one is for the return. There are several small fluctuations in the curves in graph. They could be due to rotation of the ball magnet due to the turbulence. The rotation causes the magnet to change its positive and negative poles, affecting the instant voltages. 

**Figure 9:**
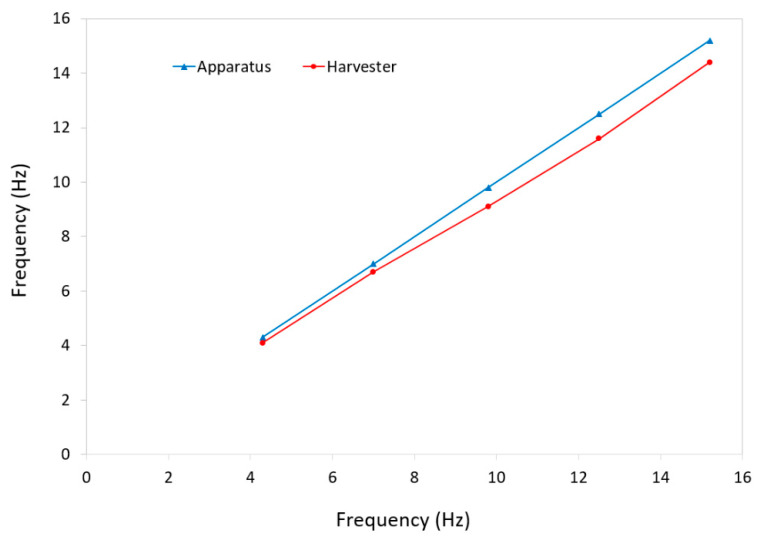
A comparison between the frequency of the testing apparatus and that of the energy harvester.

The frequency of the testing apparatus is compared with that of the energy harvester in Figure 9. The frequency of the apparatus was determined by recording the rotational speed of the machine obtained by LabVIEW and converting it to frequency while the harvester’s frequencies were calculated by determining the frequency of the generated voltages in expanded graphs, as shown in Figure 8. The results indicate that the frequency of device was closely consistent with the input frequency delivered by the testing apparatus.

The target for the application of this device was an environment at room temperature, however, one may notice that the materials used in fabrication of the materials can be slightly heated without losing their properties. PLA as the structural core material for the ball has a glass transition temperature of 65 °C which may limit the application of the ball up to this temperature, however, the outer layer of the ball, the shell is made of silicone which is among the materials with a very low thermal conductivity (~0.2 W/m·K). In addition, silicone typically has a working temperature range of 70 to 230 °C. As the shell is the outer and protecting layer of the ball, this makes the ball appropriate for higher temperature applications and its low thermal conductivity may help the ball work at even higher temperatures than PLA rating for a short amount of time. Furthermore, experiments have shown that the olive oil used as the fluid in the harvester has an exponential relationship between its viscosity and temperature. It will lose its viscosity between 0 and 40 °C and very slowly decrease over this range. The magnetic permittivity of Neodymium magnets will not change significantly up to temperatures of 65 °C. As a result, the ball may safely work at slightly higher than room temperature.

## 4. Conclusions

A multi-directional low-frequency electromagnetic energy harvester was developed. The device works based on the fluid flow when subject to a compressive force on its outer surface. It is a universal device as there is no preference on its application due to its ball shape. In addition, the device is capable of generating energy when subject to a force in any direction. The device is durable enough to withstand a long duration of use as it consists of a PLA core for its strength and a flexible silicone to absorb impacts and generate energy. The harvester was tested at various frequencies between 4 and 15 Hz and generated voltages between 17 and 44 mV. It was concluded that the harvester can generate consistent voltages at about 7.5 Hz rate.

## Figures and Tables

**Table 1:** Excitation and response frequencies and the deviation for Side C.

Excitation Frequency	Periodic Time Experimentally Measured	Response Frequency Calculated from Periodic Time	Frequency Deviation
4.3 Hz	244 ms	4.1 Hz	4.7%
7.0 Hz	149 ms	6.7 Hz	4.3%
9.8 Hz	110 ms	9.1 Hz	7.1%
12.5 Hz	86 ms	11.6 Hz	7.2%
15.2 Hz	69 ms	14.4 Hz	5.3%

## Data Availability

The datasets may be available from the corresponding author on a reasonable request.
